# Skeletal Muscle Myofibrillar Protein Abundance Is Higher in Resistance-Trained Men, and Aging in the Absence of Training May Have an Opposite Effect

**DOI:** 10.3390/sports8010007

**Published:** 2020-01-10

**Authors:** Christopher G. Vann, Paul. A. Roberson, Shelby C. Osburn, Petey W. Mumford, Matthew A. Romero, Carlton D. Fox, Johnathon H. Moore, Cody T. Haun, Darren T. Beck, Jordan R. Moon, Andreas N. Kavazis, Kaelin C. Young, Veera L. D. Badisa, Benjamin M. Mwashote, Victor Ibeanusi, Rakesh K. Singh, Michael D. Roberts

**Affiliations:** 1 School of Kinesiology, Auburn University, Auburn, AL 36849, USA; cgv0001@auburn.edu (C.G.V.); par0021@auburn.edu (P.A.R.); sco0004@auburn.edu (S.C.O.); pwm0009@auburn.edu (P.W.M.); mzr0049@auburn.edu (M.A.R.); cdf0007@auburn.edu (C.D.F.); jhm0025@auburn.edu (J.H.M.); dbeck@auburn.vcom.edu (D.T.B.); ank0012@auburn.edu (A.N.K.); kyoung@auburn.vcom.edu (K.C.Y.); 2 Department of Exercise Science, LaGrange College, LaGrange, GA 30240, USA; chaun@lagrange.edu; 3 Department of Cell Biology and Physiology, Edward Via College of Osteopathic Medicine-Auburn Campus, Auburn, AL 36832, USA; 4 United States Sports Academy, Daphne, AL 36526, USA; 5 School of the Environment, Florida A&M University, Tallahassee, FL 32306, USA; jonnada55@hotmail.com (V.L.D.B.); benjamin.mwashote@famu.edu (B.M.M.); victor.ibeanusi@famu.edu (V.I.); 6 Translational Science Lab, College of Medicine, Florida State University, Tallahassee, FL32306, USA; rakesh.singh@med.fsu.edu

**Keywords:** sarcoplasmic protein, myofibrillar protein, proteomics, aging, resistance training

## Abstract

Resistance training generally increases skeletal muscle hypertrophy, whereas aging is associated with a loss in muscle mass. Interestingly, select studies suggest that aging, as well as resistance training, may lead to a reduction in the abundance of skeletal muscle myofibrillar (or contractile) protein (per mg tissue). Proteomic interrogations have also demonstrated that aging, as well as weeks to months of resistance training, lead to appreciable alterations in the muscle proteome. Given this evidence, the purpose of this small pilot study was to examine total myofibrillar as well as total sarcoplasmic protein concentrations (per mg wet muscle) from the vastus lateralis muscle of males who were younger and resistance-trained (denoted as YT, n = 6, 25 ± 4 years old, 10 ± 3 self-reported years of training), younger and untrained (denoted as YU, n = 6, 21 ± 1 years old), and older and untrained (denoted as OU, n = 6, 62 ± 8 years old). The relative abundances of actin and myosin heavy chain (per mg tissue) were also examined using SDS-PAGE and Coomassie staining, and shotgun proteomics was used to interrogate the abundances of individual sarcoplasmic and myofibrillar proteins between cohorts. Whole-body fat-free mass (YT > YU = OU), VL thickness (YT > YU = OU), and leg extensor peak torque (YT > YU = OU) differed between groups (*p* < 0.05). Total myofibrillar protein concentrations were greater in YT versus OU (*p* = 0.005), but were not different between YT versus YU (*p* = 0.325). The abundances of actin and myosin heavy chain were greater in YT versus YU (*p* < 0.05) and OU (*p* < 0.001). Total sarcoplasmic protein concentrations were not different between groups. While proteomics indicated that marginal differences existed for individual myofibrillar and sarcoplasmic proteins between YT versus other groups, age-related differences were more prominent for myofibrillar proteins (YT = YU > OU, *p* < 0.05: 7 proteins; OU > YT = YU, *p* < 0.05: 11 proteins) and sarcoplasmic proteins (YT = YU > OU, *p* < 0.05: 8 proteins; OU > YT&YU, *p* < 0.05: 29 proteins). In summary, our data suggest that modest (~9%) myofibrillar protein packing (on a per mg muscle basis) was evident in the YT group. This study also provides further evidence to suggest that notable skeletal muscle proteome differences exist between younger and older humans. However, given that our n-sizes are low, these results only provide a preliminary phenotyping of the reported protein and proteomic variables.

## 1. Introduction

Skeletal muscle is a unique tissue in that it possesses cylindrical multi-nucleated cells with an abundance of contractile (or myofibrillar) proteins in order to facilitate muscular contractions (reviewed in [1]). Resistance training and aging are two stressors which have profound consequences on skeletal muscle physiology. For instance, several studies have demonstrated that resistance training is capable of increasing type I and II muscle fiber cross sectional areas (fCSA), which, in turn, contributes to increased tissue mass and enhances force producing capability (reviewed in [2,3,4]). On the other hand, aging in the absence of resistance training has been associated with a decrease in type II fCSA, increased fibrosis, a loss in muscle mass, and a reduction in force producing capability (reviewed in [5]).

Certain studies have reported aging [6,7], and paradoxically weeks to months of resistance training, reduce skeletal muscle myofibrillar protein density [8,9] or actin and myosin heavy chain protein abundances [10]; the latter two proteins being the predominant myofibrillar proteins. While the former occurrence is thought to be a molecular phenotype of aging [6], the latter occurrence may be reflective of *sarcoplasmic hypertrophy* where increases in muscle fiber size may occur through a more rapid expansion of the sarcoplasm relative to myofibril protein accretion [1]. Critically, this phenomenon could manifest in response to resistance training as an increase in fCSA with a concomitant decrease in either myofibrillar protein concentrations (per mg muscle) or actin and myosin heavy chain abundances (per mg tissue). While resistance training-induced sarcoplasmic hypertrophy is not a widely accepted mode of hypertrophy, there is evidence to support this construct. For instance, researchers have used transmission electron microscopy (TEM) to report that space occupied by myofibrils decreases following six months of resistance training in biceps brachii muscle fibers [8]. Other human studies have reported similar findings in the vastus lateralis using TEM [9] and biochemical methods [10]. While the functional effects of sarcoplasmic hypertrophy remained to be determined, we speculate that the purpose of such a mechanism is to spatially and bioenergetically prime muscle cells for eventual myofibril expansion. Notwithstanding, and assuming sarcoplasmic hypertrophy is a mechanistic outcome of shorter-term training, it remains to be determined if years of resistance training produces molecular features indicative of this phenomenon.

Proteomics have allowed scientists to examine the relative expression of individual skeletal muscle proteins of younger versus older participants [7,11] as well as in younger participants prior to and following resistance training [10,12]. Along with studies reporting that older participants have lower actin and myosin heavy chain abundances as described above, these proteomic investigations have indicated that older participants: (a) express a muscle proteomic profile indicative of enhanced oxidative capacity and reduced glycolytic capacity [7], and (b) demonstrate a fiber type-specific dysregulation in the expression of metabolic enzymes [11]. Regarding the proteomic interrogation of skeletal muscle in response to resistance training, Hody et al. [12] reported that two weeks of eccentric leg extensor resistance training decreased the relative expression of various contractile proteins. Additionally, we recently reported that six weeks of high volume resistance training significantly increased mean muscle fiber cross sectional area (fCSA) in 15 of 31 previously-trained college-aged males, although the relative abundances of myosin heavy chain and actin decreased, and numerous sarcoplasmic proteins related to glycolysis and ATP generation increased in these individuals [10]. While in rats, another recent study suggested that higher-volume ladder climbing decreased the abundances of several myofibrillar proteins in lieu muscle fCSA increases, whereas these alterations were not evident with lower-volume training [13]. Thus, in agreement with the previously discussed data, these proteomic investigations suggest that sarcoplasmic hypertrophy may arise from weeks to months of higher-volume resistance training.

Given the supporting literature cited above, we posit that years of resistance training-induced skeletal muscle growth may be partially modulated through sarcoplasmic hypertrophy. Therefore, the purpose of this small pilot study was to examine the body composition, muscle fiber sizes, and total myofibrillar as well as total sarcoplasmic protein concentrations from the vastus lateralis muscle of young resistance-trained men who self-reported consistently weight lifting for an average of 10 years (YT) and young untrained men (YU). Given that we had access to body composition data along with stored skeletal muscle from an older non-resistance-trained male cohort (OU), we also sought to compare these variables amongst the YT, YU, and OU cohorts. We hypothesized that YT participants would present greater hypertrophic indices (i.e., fat-free mass index, vastus lateralis thickness, mean muscle fCSA) compared to the YU and OU cohorts. However, in line with our speculations regarding potential sarcoplasmic hypertrophy, we hypothesized the YT group would present lower total myofibrillar protein concentrations (per mg tissue), lower abundances of actin and myosin heavy chain (per mg tissue), and greater total sarcoplasmic protein concentrations (per mg tissue) compared to YU and OU cohorts.

## 2. Materials and Methods

### 2.1. Ethical Approval and Study Design

Participants were involved in two studies approved by the Institutional Review Board at Auburn University (Protocol #18-442 MR 1812, Protocol #18-226 AR 1806; IRB contact: irbadmin@auburn.edu). Inclusion criteria indicated that participants had to be male and abstain from nutritional supplementation for one month prior to the study (with the exception of multivitamins). Participants had to be free of cardio-metabolic diseases (e.g., morbid obesity, type II diabetes, severe hypertension) or conditions which precluded the collection of a skeletal muscle biopsy. Additionally, participants could not be on medications to treat metabolic conditions (e.g., cholesterol medications, blood pressure medications, medications to ameliorate high blood sugar). All participants provided verbal and written consent to participate in each respective study, and both studies conformed to the standards set by the latest revision of the Declaration of Helsinki. Self-reported training status was collected through written questionnaires prior to the testing session described below. The YT group (n = 6) possessed an average age of 25 ± 4 years, the YU group (n = 6) possessed an average age of 21 ± 1 years, and the OU group (n = 6) possessed an average age of 62 ± 8 years (OU > YT&YU, *p* < 0.001 at each comparison; YT > YU, *p* = 0.025). The YT group had a self-reported resistance training age of 10 ± 3 years (range: 6–15 years), the YU group had a self-reported training age of <1 year, and the OU group had a self-reported training age of 0 years within the past decade (YT > YU&YO, *p* < 0.001 at each comparison).

### 2.2. Testing Session

The testing session below occurred during the morning hours (05:00–09:00) following an overnight fast, and procedures are described in the order that they occurred.

#### 2.2.1. Body Composition and Hydration Testing

Prior to testing batteries, participants submitted a urine sample (~5 mL) to assess urine specific gravity (USG) levels using a handheld refractometer (ATAGO; Bellevue, WA, USA). Notably, USG levels in all participants were ≤1.020. Body composition was measured by bioelectrical impedance spectroscopy (BIS) using methods previously described (SOZO Device, ImpediMed, Limited, Brisbane, Qld, Australia) [14]. This method has been shown to produce test–retest intraclass correlation coefficients (ICC) >0.990 for whole body fat mass, fat-free mass, and percent body fat in 29 men and women with comparable results to dual-energy X-ray absorptiometry in 95 men and women [14]. Additionally, the BIS method used has been identified as an acceptable alternative to dual-energy X-ray absorptiometry for use in clinical practice for body composition analyses [14].

#### 2.2.2. Ultrasound for Vastus Lateralis (VL) Thickness

Participants had the right leg VL muscle subjected to ultrasound using a 3–12 MHz multi-frequency linear phase array transducer (Logiq S7 R2 Expert; General Electric, Fairfield, CT, USA) to determine muscle thickness. Participants were instructed to stand and displace bodyweight more to the left leg to ensure the right leg was relaxed. Measurements were standardized by placing the transducer at the midway point between the iliac crest and patella. Ultrasounds were performed by the same technician. In a prior study from our laboratory, this investigator produced a test–retest ICC of 0.994 on 33 participants [15].

*Muscle tissue collection.* Right leg VL muscle biopsies were obtained with a 5-gauge needle as previously described by our laboratory [15]. Following biopsies, ~20–40 mg of tissue was embedded in optimal cutting temperature (OCT) media (Tissue-Tek, Sakura Finetek Inc; Torrence, CA, USA), as previously described by our laboratory [16]. Remaining tissue was teased of blood and connective tissue, and subsequently stored at −80 °C for further molecular analyses.

#### 2.2.3. Leg Extensor Peak Torque Testing Using an Isokinetic Dynamometer

For right leg extensor peak torque testing, participants were fastened to an isokinetic dynamometer (Biodex System 4, Biodex Medical Systems, Inc., Shirley, NY, USA). Each participant’s lateral epicondyle was aligned with the axis of the dynamometer, and seat height was adjusted to ensure the hip angle was approximately 90°. Prior to torque assessment, each participant performed a warmup consisting of submaximal to maximal isokinetic knee extensions. Participants then completed five maximal voluntary isokinetic knee extension actions at 2.09 rad/s (120°/s). Participants were provided verbal encouragement during each contraction. The isokinetic contraction resulting in the greatest value was used for analyses. Notably, all YT and 3/6 YU participants performed this test roughly 30 min following all of the procedures in the testing session above, whereas the other participants performed this test approximately one week prior to the testing session above. Notwithstanding, all participants were fasted for at least four hours prior to this test.

### 2.3. Biochemical Assays

#### 2.3.1. Sarcoplasmic and Myofibrillar Protein Isolation

Sarcoplasmic and myofibrillar protein isolations were performed per our laboratory as previously described with minor modifications [10,17]. Briefly, skeletal muscle foils were removed from −80 °C, placed on a liquid nitrogen-cooled ceramic mortar and pestle, and muscle was powdered. Pulverized tissue from each sample (~20 mg) was weighed on an laboratory scale exhibiting a sensitivity of 0.0001 g (Mettler-Toledo; Columbus, OH, USA) and quickly placed in 200 μL ice-cold buffer (Buffer 1: 25 mM Tris, pH 7.2, 0.5% Triton X-100, protease and phosphatase inhibitors). Samples were homogenized using tight-fitting pestles and centrifuged at 1500× *g* for 10 min at 4 °C. Supernatants (sarcoplasmic fraction) were collected and placed in new 1.7 mL microtubes on ice. As a wash step, the resultant myofibrillar pellet was resuspended in Buffer 1 and centrifuged at 1500× g for 10 min at 4 °C. The supernatant was discarded and the myofibrillar pellet was solubilized in 300 μL of ice-cold storage buffer (20 mM Tris-HCl, pH 7.2, 100 mM KCl, 20% glycerol, 1 mM DTT, 50 µM spermidine, protease and phosphatase inhibitors). The sarcoplasmic and myofibrillar resuspensions were stored at −80 °C until protein concentration determination and proteomic analyses described below.

#### 2.3.2. Determination of Protein Concentrations

Sarcoplasmic and myofibrillar protein fractions obtained above were batch-assayed for protein concentration determination using a commercially-available bicinchoninic acid (BCA) kit (Thermo Fisher Scientific; Waltham, MA, USA). Samples were assayed in triplicate using a microplate assay protocol (20 μL of 5× diluted sample + 200 μL Reagent A + B). Triplicate coefficient of variations (CVs) for total sarcoplasmic and total myofibrillar protein concentrations were 5.0% and 5.4%, respectively. To derive final total sarcoplasmic protein concentrations, BCA-yielded concentrations were multiplied by the amount of Buffer 1 used (200 μL) and divided by the amount of wet muscle used for the assay. To derive final total myofibrillar protein concentrations, BCA-yielded concentrations were multiplied by the amount of buffer used to resuspend the pellet (300 μL) and divided by the amount of wet muscle used for the assay.

The myofibrillar resuspensions were subsequently assayed for myosin heavy chain and actin protein abundances using SDS-PAGE and Coomassie staining, and both fractions were analyzed using proteomics described below.

#### 2.3.3. SDS-PAGE and Coomassie Staining for Determination of Relative Myosin Heavy Chain and Actin Abundances

Actin and myosin heavy chain protein abundances were assessed using SDS-PAGE as previously described by our laboratory [10,17] and others [18]. Briefly, samples were prepared for SDS-PAGE using 10 μL resuspended myofibrils, 65 μL distilled water (diH_2_O), and 25 μL 4× Laemmli buffer. Samples (5 μL) were placed on gradient SDS-polyacrylamide gels (4%–15%) in duplicate (Bio-Rad Laboratories), and electrophoresis commenced at 200 V for 40 min. Thereafter, gels were washed in diH_2_O for 15 min and stained for 2 h using a modified Coomassie reagent (LabSafe GEL Blue; G-Biosciences; St. Louis, MO, USA). Gels were then destained in diH_2_O for 60 min, imaged with a digital camera (iPhone 8; Cupertino, CA, USA), and band densities were assessed with a gel documentation system (UVP). Since standardized volumes for samples were loaded on SDS-PAGE gels, actin and myosin heavy chain band densities were normalized to input muscle weights to derive relative abundances (or arbitrary density units (ADU)) per mg wet muscle. Duplicate coefficient of variations (CVs) for actin and myosin heavy chain band densities were 1.5% and 1.3%, respectively.

#### 2.3.4. Proteomic Analysis of the Sarcoplasmic and Myofibrillar Fractions

Proteomics were performed on the sarcoplasmic and myofibrillar protein fractions similar to previous work published by our laboratory [10]; please refer to this publication for detailed methods. Previous data from our laboratory have indicated that triplicate CVs for all targets interrogated via proteomics has not generally exceeded 10% [10].

As part of our analysis for the current project, we manually interrogated proteomics data for the following prominent myofibrillar proteins to examine differences between cohorts: (a) myosin heavy chain (summed isoforms which included MYH1/2/3/4/7/8/9/11/13/16), (b) actin (summed isoforms which included ACTA1, ACTC1, ACTG1), (c) troponin (summed isoforms which included TNNC1, TNNC2, TNNI1, TNNI2, TNNT1, TNNT2), (d) titin, (e) tropomyosin (summed isoforms which included TPM1, TPM2, TPM3), (f) alpha-actinin (summed isoforms which included ACTN1/2/3), and (g) nebulin. Notably, these targets were derived from our recent review where we discussed these proteins making up greater than 90% of the myofibrillar protein fraction [1].

Based on the “cellular process” classifications provided by Scaffold v4.0, we also manually interrogated proteomic data for following prominent sarcoplasmic proteins (or protein clusters) to examine differences between cohorts: (a) prominent glycolysis enzymes (cellular process: “canonical glycolysis”, which included AGL, ALDOA/C, ENO1/2/3, FBP2, GALM, GAPDH, GDP1, GPI, GYG1, GYS1, HK1, LDHA/B, MPI, PFKM, PGAM1/2, PGK1, PGM1, PGP, PHKA1, PHKB, PHKG1, PKM, PYGM, and TPI1), (b) creatine kinase (summed isoforms which included CKM, CKB, CKMT2), (c) prominent Krebs cycle enzymes (cellular process: “tricarboxylic cycle”, which included ACO1/2, MDH1/2, IDH1, IDH3A/G/B, IDH2, CS, CYC1, CYCS, FH, SUCLA2, SUCLG1/2, OGDH, PDHA1, PDHB, PDHX), (d) prominent beta-oxidation enzymes (cellular process: “fatty acid beta-oxidation”, which included ACAA2, ACADM, ACADS, ACADSB, ACAT1, ACOT1, ACSL1, ECHS1, ECI1, DBI, ETFA, ETFB, ETFDH, GPD1L, GPD2, HADH), (e) prominent electron transport chain proteins (cellular process: “mitochondrial electron transport”, which included: ATP5F1A/B, ATP5PD, ATP5F1C/D, ATP5ME/F/G, ATP5PB/F/O, COX4I1, COX5A/B, COX6B1, COX6C, COX7A1, CYB5R1/3, MT-CO2, MT-ND5, NDUFA2/4/5/6/7/8/9/10/12/13, NDUFAB1, NDUFB3/4/6/8/10/11, NDUFC2, NDUFS1/2/3/5/6/7/8, NDUFV1/2, SDHA, SDHC, UQCRB, UQCRC1, UQCRFS1, UQCRH).

#### 2.3.5. fCSA Analysis

Methods for immunohistochemistry were employed as previously reported by our laboratory and described elsewhere [16,19,20]. Once all samples were sectioned, sections were batch-processed for immunohistochemistry. Briefly, sections were air-dried at room temperature for 10 min and blocked with 100% Pierce Super Blocker (Thermo Fisher Scientific) for 25 min. Sections were then incubated with a pre-diluted commercially-available rabbit anti-dystrophin IgG antibody solution for 60 min (catalog #: GTX15277; Genetex Inc.; Irvine, CA, USA), and this solution was spiked with mouse anti-myosin I IgG (catalog #: A4.951 supernatant; Hybridoma Bank, Iowa City, IA, USA; 40 µL added per 1 mL of dystrophin antibody solution). Sections were then washed for 2 min in PBS and incubated for 60 min with a secondary antibody solution containing Texas Red-conjugated anti-rabbit IgG (catalog #: TI-1000; Vector Laboratories, Burlingame, CA, USA) and Alexa Fluor 488-conjugated anti-mouse IgG (catalog #: A-11001; Thermo Fisher Scientific) (~6.6 µL of all secondary antibodies per 1 mL of blocking solution). Sections were washed for 5 min in PBS, air-dried, and mounted with fluorescent media containing 4,6-diamidino-2-phenylindole (DAPI; catalog #: GTX16206; Genetex Inc.). Sections were mounted, and digital images were immediately captured with a fluorescent microscope (Nikon Instruments, Melville, NY, USA) using a 10× objective. TRITC and FITC images were captured at 600 ms exposures, and DAPI images were captured at 80 ms exposures. This method identified cell membranes (Texas Red filter), type I fibers (FITC filter), type II fibers (unlabeled), and myonuclei (DAPI filter). Measurements of fCSA were performed using the open-sourced software MyoVision [21]. A pixel conversion ratio value of 0.493 was used to account for the size and bit-depth of images, and a detection range of detection from 200 to 12,000 μm^2^ was used to ensure artifact was removed (e.g., small “false” fibers or large fibers which may have not been in transverse orientation).

### 2.4. Statistical Analysis

All statistical analyses were performed using SPSS v22.0 (IBM Corp, Armonk, NY, USA). Shapiro-Wilk tests of normality indicated that all variables except type II fCSA and mean fCSA were normally distributed. Thus, normally distributed variables were analyzed using one-way ANOVAs with LSD post hoc tests, whereas type II fCSA and mean fCSA were square root-transformed prior to analysis. All proteomics data were analyzed using were analyzed using one-way ANOVAs with LSD post hoc tests. Pearson correlations were also performed on select variables. Data are presented throughout as means ± standard deviation (SD) values and statistical significance was established as *p* < 0.050.

## 3. Results

### 3.1. Participant Characteristics

**Figure 1:**
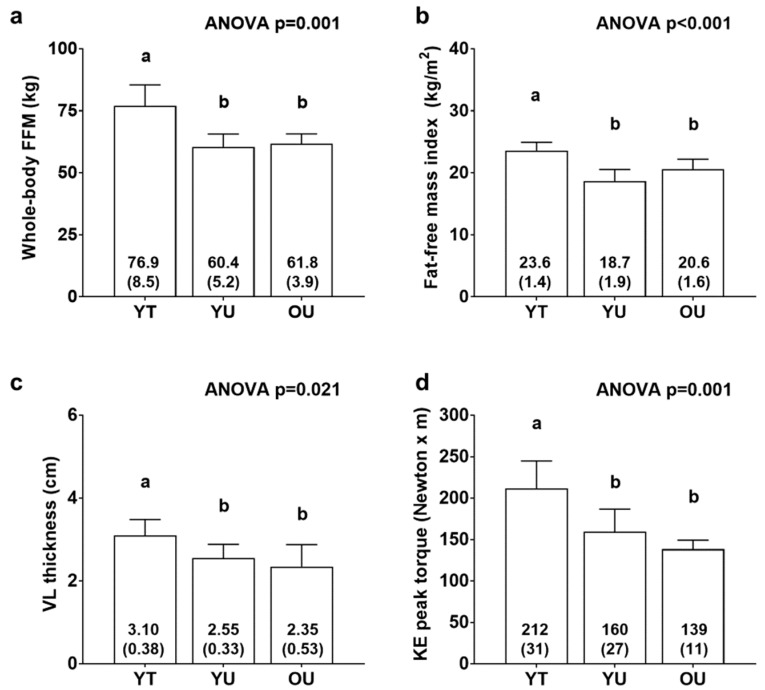
Body composition and knee extensor torque differences between cohorts. Data include whole-body fat-free mass (FFM) (panel **a**), fat-free mass index (panel **b**), vastus lateralis thickness (panel **c**), and knee extensor (KE) peak torque (panel **d**) of younger trained (YT), younger untrained (YU), and older untrained (OU) participants. Numerical values within bars represent mean ± standard deviation values. Bars with different superscript letters indicate a significant difference between cohorts (*p* < 0.05).

Figure 1 contains body composition and knee extensor torque data. There were significant differences between cohorts for FFM (YT > YU = OU, *p* < 0.01; Figure 1a), fat-free mass index (YT > YU = OU, *p* < 0.01; Figure 1b), VL thickness (YT > YU = OU, *p* < 0.05; Figure 1c), and knee extensor peak torque (YT > YU = OU, *p* < 0.05; Figure 1d). There was also differences between groups for total body mass (YT: 97.9 ± 11.8 kg, YU: 78.9 ± 8.2 kg, OU: 76.6 ± 2.2 kg; YT > YU = OU, *p* < 0.05, data not shown) and fat mass (YT: 21.0 ± 4.7 kg, YU: 18.5 ± 6.6 kg, OU: 14.8 ± 3.3 kg; YT > OU, *p* < 0.05, data not shown). However, there were no differences between groups for percent body fat (YT: 21.3 ± 3.1 %, YU: 23.1 ± 6.7 %, OU: 19.4 ± 4.4 %, data not shown).

**Figure 2:**
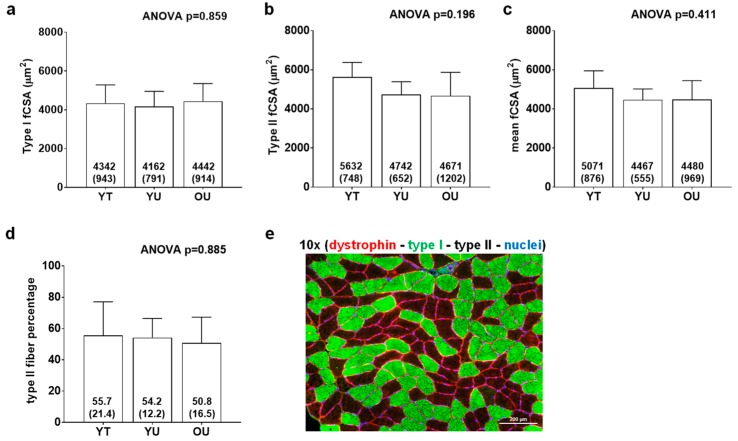
Histology data between cohorts. Data include type I fiber cross-sectional area (fCSA) (panel **a**), type II fCSA (panel **b**), mean (type I + II) fCSA (panel **c**), and type II fiber percentage (panel **d**) of younger trained (YT), younger untrained (YU), and older untrained (OU) participants. Numerical values within bars represent mean ± standard deviation values. Panel **e** contains a representative 10× image of muscle fibers in cross section (scale bar = 200 μm).

Figure 2 contains histology data. There were no differences between groups for type I fCSA (ANOVA *p* = 0.859), mean fCSA (ANOVA *p* = 0.160), or type II fiber percentage (ANOVA *p* = 0.885). On average, 124 ± 31 fibers were quantified across participants.

### 3.2. Total Myofibrillar and Sarcoplasmic Protein Concentrations

**Figure 3:**
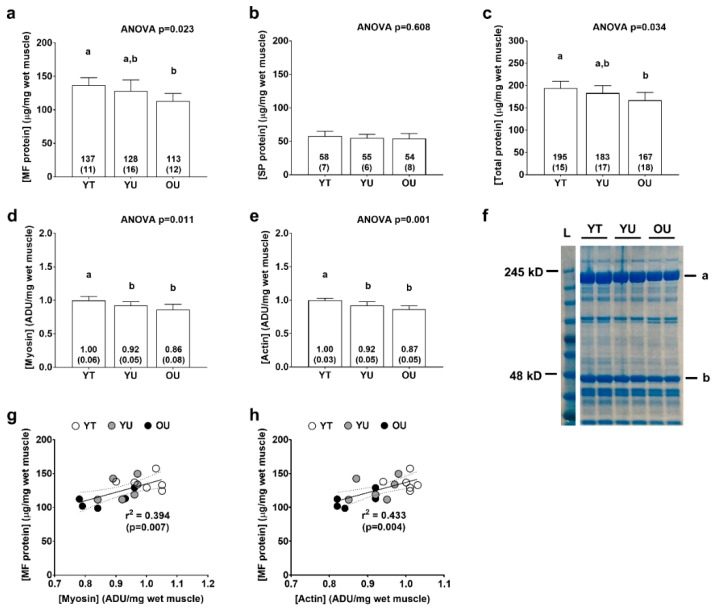
Total myofibrillar, myosin, actin, and sarcoplasmic protein concentrations. Data include total myofibrillar (MF) protein concentrations (panel **a**), total sarcoplasmic protein (SP) concentrations (panel **b**), total protein concentrations (panel **c**), myosin heavy chain protein abundance (panel **d**), and actin protein abundance (panel **e**) of younger trained (YT), younger untrained (YU), and older untrained (OU) participants. Numerical values within bars represent mean ± standard deviation values. Panel **f** contains a representative Coomassie image of a participant from each cohort (duplicate lanes) where myosin heavy chain is represented by band (**a**), and actin by band (**b**). Panels (**g,h**) are regression plots with 95% confidence intervals showing good agreement between actin and myosin heavy chain abundance (determined via SDS-PAGE) versus total myofibrillar protein concentrations (per mg tissue) (determined using BCA assays).

There were significant differences between groups for total myofibrillar protein concentrations (YT > OU, *p* = 0.005; Figure 3a) and total protein (myofibrillar + sarcoplasmic) concentrations (YT > OU, *p* = 0.015; Figure 3c), but not total sarcoplasmic protein concentrations (ANOVA *p* = 0.608; Figure 3b). There were also significant differences between groups for myosin heavy chain protein abundance (YT > YU = OU, *p* < 0.05 at each comparison; Figure 3d) and actin protein abundance (YT > YU = OU, *p* < 0.05; Figure 3e) as determined by SDS-PAGE. When all participants were pooled, there were significant associations between total myofibrillar protein concentrations and myosin heavy chain abundance (r^2^ = 0.394, *p* = 0.007; Figure 3g) as well as total myofibrillar protein concentrations and actin abundance (r^2^ = 0.433, *p* = 0.004; Figure 3h) suggesting that the isolated myofibrillar protein pools was largely made up of these two proteins.

### 3.3. Muscle Proteome Analysis

A total of 810 muscle proteins were identified using proteomics. With proteome analysis, we were interested in determining four potential relationships including: (a) myofibrillar proteins which demonstrated a potential long-term training effect (YT><YU&OU, *p* < 0.05), (b) sarcoplasmic proteins which demonstrated a potential long-term training effect (YT><YU&OU, *p* < 0.05), (c) myofibrillar proteins which demonstrated an age effect (OU><YT&YU, *p* < 0.05), and (d) sarcoplasmic proteins which demonstrated an age effect (OU><YT&YU, *p* < 0.05).

Table 1 shows proteins in the myofibrillar fraction which demonstrated a potential long-term training effect. Five proteins were lower in YT participants versus participants in the other groups, whereas one protein was greater in YT participants versus participants in the other groups.

Table 2 shows proteins in the sarcoplasmic fraction which demonstrated a potential long-term training effect. One protein was lower in YT participants versus participants in the other groups, whereas five proteins were greater in YT participants versus participants in the other groups.

Table 3 shows proteins in the myofibrillar fraction which demonstrated an aging effect. Seven proteins were lower in OU participants versus participants in the other groups, whereas 11 proteins were greater in OU participants versus participants in the other groups.

Table 4 shows proteins in the sarcoplasmic fraction which demonstrated an aging effect. Eight proteins were lower in OU participants versus participants in the other groups, whereas 29 proteins were greater in OU participants versus participants in the other groups.

### 3.4. Extrapolation of Muscle Composition between Groups

**Figure 4:**
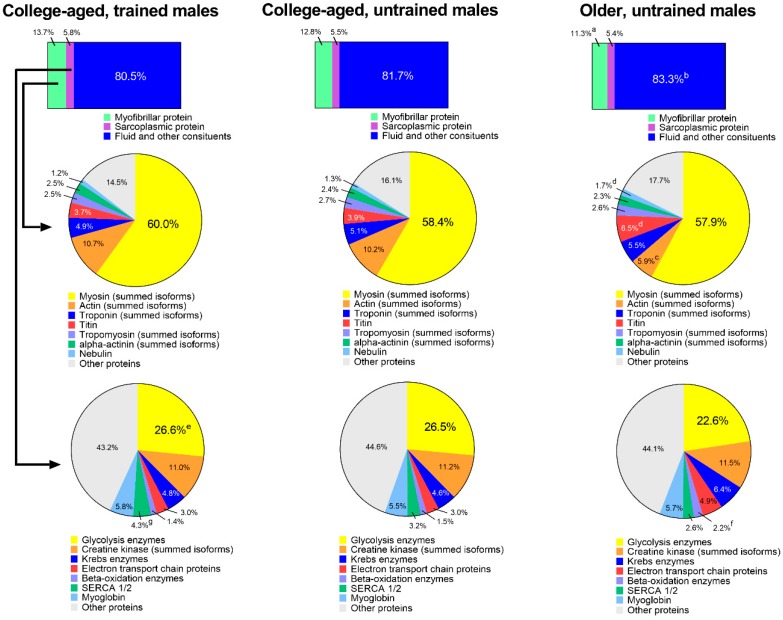
Estimated muscle compositional differences between groups. Top bar insets were derived from bicinchoninic acid (BCA) protein assays, middle pie charts represent the percent contribution of different contractile proteins to the myofibrillar pool derived from proteomics, and bottom pie charts represent the percent contribution of different proteins to the sarcoplasmic pool derived from proteomics. Symbols: (**a**) indicates that the contribution of myofibrillar protein to tissue mass was greater in YT versus OU (*p* < 0.05); **b**, indicates that the contribution of fluid and other constituents to tissue mass was greater in OU versus YT (*p* < 0.05); (**c**) indicates that the contribution of actin to the myofibrillar protein pool was greater in YT and YU versus OU (*p* < 0.05); (**d**) indicates that the contributions of titin and nebulin to the myofibrillar protein pool were greater in OU versus YT and YU (*p* < 0.05); **e**, indicates that the contribution of glycolysis enzymes to the sarcoplasmic protein pool was greater in YT versus OU (*p* < 0.05); **f**, indicates that the contribution of beta-oxidation enzymes to the sarcoplasmic protein pool was greater in OU versus YT and YU (*p* < 0.05); (**g**) indicates that the contribution of SERCA1/2 to the sarcoplasmic protein pool was greater in YT versus OU (*p* < 0.05).

Figure 4 shows an extrapolation of muscle protein composition between groups. When data were expressed as percent contribution to wet muscle weight (µg myofibrillar protein/µg wet muscle weight), YT participants possessed 13.7 ± 1.1% myofibrillar protein, YU participants possessed 12.8 ± 1.6%, and OU participants possessed 11.3 ± 1.2% (YT > OU, *p* = 0.015). Percent contribution of sarcoplasmic protein to wet muscle weight was equal between groups, although percent contribution of fluid and other constituents (i.e., everything other than muscle proteins) was greater in OU versus YT participants (83.3 ± 1.8% versus 80.5 ± 1.5%, respectively; *p* = 0.015).

The first row of pie charts depict the percent contribution of prominent myofibrillar proteins to the total myofibrillar protein fraction. There were no differences between groups for myosin heavy chain (summed isoforms), troponin (summed isoforms), tropomyosin (summed isoforms), or alpha-actinin (summed) isoforms. Actin (summed isoforms) was significantly lower in OU participants versus participants in both younger groups (*p* < 0.05), and titin and nebulin were significantly higher in OU participants versus participants in younger groups (*p* < 0.05).

The second row of pie charts show the percent contribution of prominent sarcoplasmic proteins to the total sarcoplasmic protein fraction. There were no differences between groups for creatine kinase (summed isoforms), Krebs enzymes, electron transport chain proteins, or myoglobin. The expression of glycolysis enzymes (26.6 ± 1.2% versus 22.6 ± 3.2%, *p* = 0.017) and SERCA1/2 (4.3 ± 0.7% versus 2.6 ± 0.6%, *p* = 0.002) were greater in YT versus OU participants, and the contribution of beta-oxidation enzymes were greater in OU (2.2 ± 0.7%) versus YT (1.4 ± 0.1%, *p* = 0.025) and YU participants (1.5 ± 0.4%, *p* = 0.048).

## 4. Discussion

The purpose of this small pilot study was to examine total myofibrillar as well as total sarcoplasmic protein abundances from the vastus lateralis muscle of young resistance-trained, young untrained, and older non-resistance-trained men. Additionally, the abundances of actin and myosin heavy chain (per mg tissue) were examined using SDS-PAGE and Coomassie staining, and shotgun proteomics was used to interrogate individual protein differences in each fraction between cohorts. YT participants exhibited greater hypertrophic indices compared to the YU and OU groups (Figure 1). YT participants also possessed greater total myofibrillar protein concentrations compared to OU participants, and YT participants possessed greater actin and myosin heavy chain abundances as well as similar total sarcoplasmic protein concentrations compared to the YU and OU groups (Figure 3). Contrary to our hypothesis, these findings suggest long-term resistance training may not result in sarcoplasmic hypertrophy. Rather, years of resistance training may promote marginal increases in *myofibrillar protein packing* as evidenced by ~9% greater actin and myosin heavy chain abundances in the YT versus YU group. For the sake of clarity, myofibrillar protein packing is the presence of more actin, myosin heavy chain, and other sarcomeric proteins (per mg tissue). This phenomenon likely occurs through either the addition of proteins to existing myofibrils, or the construction of new myofibrils [22]. While our findings are compelling, more research is needed before confidently defending this thesis due the limited sample size herein.

**Figure 5:**
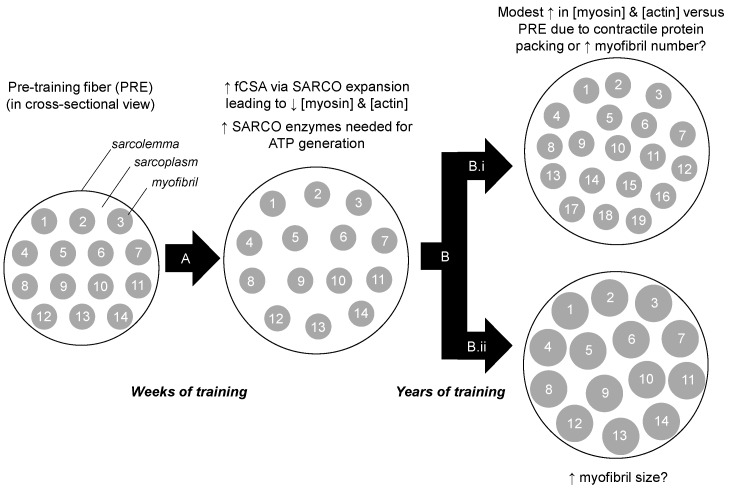
Theoretical model of training-induced myofibrillar protein adaptations. Legend: The left portion of the figure shows a muscle fiber in cross section. After weeks to months of resistance training (**A**), we hypothesize that there is likely an increase in cell size due to sarcoplasmic (SARCO) expansion. Additionally, we posit that many sarcoplasmic enzymes are up-regulated to generate ATP in order to cope with energy demands during exercise. Our current data suggest that after years of resistance training (**B**) there may be modest packing of contractile proteins. Whether this manifests through an increase in myofibril number (**B.i**), or myofibril size (**B.ii**), remains to be determined through advanced histological interrogations.

Our findings contrast other studies. For instance, long-term resistance training has been reported to reduce intramuscular myofibrillar volume [8], and another study indicated specific tensions are lower in muscle fibers isolated from bodybuilders compared to fibers isolated from power athletes and control subjects [23]. The latter study in particular suggests that years of training decreases myofibril density considering that the myofibril is the site of force production. It is notable, however, that critical differences exist between the current study and these previous studies. Specifically, nine of the 12 bodybuilders in the Meijer et al. study, and six of the seven powerlifters in the MacDougall et al. study, reported using anabolic steroids. While none of the YT participants self-reported using anabolic steroids herein, we elected to perform serum analysis using a total testosterone EIA-based assay (Alpco Laboratories) given that this potential confounder was a concern to our outcome measures. The assay indicated that YT participants presented total testosterone values that were within a normal physiological range (487 ± 49 ng/dL, range 412–543 ng/dL), and substantially lower than values previously reported in college-aged males which were injected with recreational doses of testosterone enanthate (>3200 ng/dL) [24]. Thus, we speculate sarcoplasmic hypertrophy observed in both of the aforementioned studies was likely a result of supraphysiological hypertrophy facilitated through anabolic steroid use. Beyond the two aforementioned long-term training studies, it is notable that we previously reported six weeks of very high volumes resistance training increased markers of sarcoplasmic hypertrophy in previously-trained participants [10]. However, we speculate that this may be a high volume training-induced mechanism through which muscle cells spatially prime themselves for the eventual accretion of myofibrillar proteins. Alternatively stated, we find it unlikely that the YT participants were utilizing training loads prior to tissue sampling similar to what were employed in our prior study. In lieu of this collective evidence, we propose a model of sarcoplasmic hypertrophy primarily from short-term, high volume training during which muscle fibers are spatially and metabolically primed for subsequent myofibrillar hypertrophy from continued training (Figure 5). Notably, this model opposes Phillips’ model of resistance training-induced hypertrophy where the author hypothesizes that myofibrillar packing precedes fCSA expansion within the first 8 weeks of training [22]. Thus, given these divergent hypotheses, more research in this area is needed.

If resistance training-induced sarcoplasmic hypertrophy is indeed a training adaptation, future studies should determine how training volume and load affect this process. There is recent evidence to suggest that lactate, which accumulates in skeletal muscle from higher volume training, is capable of stimulating muscle protein synthesis and hypertrophy in myotubes (in vitro) as well as rodents [25]. It may be possible that lactate accumulation from high volume training preferentially stimulates the fractional synthetic rates of metabolic enzymes related to glycolysis, thus leading to greater sarcoplasmic hypertrophy, whereas high load training may equally stimulate the fractional synthetic rate of sarcoplasmic as well as contractile proteins.

### 4.1. Proteome Differences between YT versus Other Groups

According to our proteomics analysis, there were only marginal differences in the expression of individual myofibrillar or sarcoplasmic proteins between the YT versus YU and OU groups which precluded formal bioinformatics analyses. Nonetheless, there were interesting differences in select protein targets. For instance, the dystrophin (DMD) protein in the myofibrillar fraction was lowest in YT participants and highest in OU participants. This is consistent with a previous report which has demonstrated that resistance exercise-induced muscle damage downregulates DMD protein levels in rats [26]. However, others have reported that eight weeks of resistance training does not affect muscle DMD protein levels in previously untrained human male participants [27]. Comparing results from the latter study to the current dataset is limited given that the YT participants in this study consistently trained for nearly 10 years. Thus, whether the DMD downregulation occurred with years of resistance training, as well as the functional ramifications of this downregulation, remains unknown and should be further explored. Higher levels of sarcoplasmic phosphorylase b kinase gamma catalytic chain (PHKG1) as well as annexin A6 (ANXA6) in the YT participants are congruent with what would be anticipated from long-term resistance training. With regard to the former target, it has been demonstrated that one bout of resistance exercise substantially increases intramuscular glycolysis metabolites [28], and we and others have reported that weeks of resistance training increases the expression of enzymes related to glycolytic flux [10,29]. Hence, higher protein levels of PHKG1 in the YT participants fit this paradigm. The *Anxa6* gene has been shown to be critical in mice for muscle cell membrane repair [30,31]. Given that resistance exercise acutely damages the sarcolemma [26], it is sensible that an upregulation in the ANXA6 protein is likely involved in the recovery-adaptation response to resistance training. Beyond these marginal proteomic differences, however, the overall lack of differences in the muscle proteome between the YT group and other two groups suggests that global skeletal muscle protein expression patterns remain largely unaffected with years of resistance training.

### 4.2. Proteome Differences between OU versus Other Groups

Some studies suggest that the abundance of individual myofibrillar proteins are lower, whereas the abundance of oxidative enzymes is higher in skeletal muscle from older versus younger individuals [32]. Several proteins in the myofibrillar fraction were differentially expressed between the OU versus younger groups. The lower actin protein abundance in the older participants agrees with other studies which have shown that total myofibrillar protein concentrations [6], as well as the abundances of specific contractile proteins [7], are lower in older versus younger individuals. However, older participants exhibited higher levels of titin, nebulin, troponin T, and dystrophin in the myofibrillar fraction relative to younger participants. While this finding is difficult to reconcile, it is notable that previous proteomic studies have reported that older humans [7] and rodents [33] express higher levels of certain contractile proteins relative to their younger counterparts.

The sarcoplasmic protein pool was found to be most affected with aging and, because there were numerous sarcoplasmic protein abundances that differed between older versus younger participants (Table 4), we elected to perform formal bioinformatics analyses. According to KEGG analysis [34], pathways that were significantly upregulated in older participants included “metabolic pathways” (13 proteins OU > other groups; Benjamini *p* = 0.0004), “butanoate metabolism” (4 proteins OU > other groups; Benjamini *p* = 0.0019), “fatty acid degradation” (4 proteins OU > other groups; Benjamini *p* = 0.0048), “fatty acid elongation” (3 proteins OU > other groups; Benjamini *p* = 0.035), and “carbon metabolism” (4 proteins OU > other groups; Benjamini *p* = 0.042). These pathways collectively suggest that aspects of oxidative metabolism are greater in muscle from older participants, and this age-related phenomenon has been previously reported [32]. It is also interesting to note that cathepsin D (CTSD), which is involved with lysosomal proteolysis [35], was greatest in older participants, and that proteins involved with other proteolysis pathways were not affected; specifically proteins involved with the ubiquitin proteasome (UBE2V2, USP14, UBE2L3, USP5, UBE2N, PSMA1, PSMA3, PSMA6, PSMA7, PSMB1, PSMB4, PSMB7, PSMD2, PSME1) or calpain pathways (CAPN1, CAPN2, CAPN3) were not differently expressed between age groups. These data agree with previous reports which showed that cathepsin D is upregulated in older human [36] and rodent skeletal muscle [37], and this may be an age-related atrophy target that should be further interrogated.

### 4.3. Experimental Considerations

This study is limited given that it was a cross-sectional analysis with a small number of male participants per group. In this regard, results from this small pilot study only serve to provide a preliminary phenotype of the outcome variables between the interrogated groups, and more research is needed to verify our findings. Another limitation to the current study is that, while not well-trained, most of the older participants self-reported routinely engaging in activities such as walking, jogging or bike-riding. This likely explains why some of the outcome measures were not different between the YU and OU groups (e.g., FFM, VL thickness, and percent body fat), and re-iterates that data from the OU group are not reflective of sedentary, frail older individuals. The older individuals were also more varied in age relative to the younger groups (age range: 52–71 years old). Thus, this could have led to some variability in the outcome measures. We also elected to implement LSD post hoc tests a priori which are relatively liberal compared to other post hoc tests. Thus, our findings should be interpreted in this regard. Finally, while the YT group had more resistance training experience than the other groups per the self-reported data gathered along with data in Figure 1, we did not collect detailed resistance training records of these participants. Critically, some of these individuals may have recently subscribed to different training methods versus other individuals (e.g., high load versus high volume training). Likewise, some individuals reported more training years versus others (e.g., 6 years versus 15 years). Both of these attributes may have affected outcomes in this study and remain as unresolved limitations.

## 5. Conclusions

Contrary to our hypothesis, long-term resistance training may in fact lead to a marginal degree of contractile protein packing as evidenced with the greatest myosin heavy chain and actin protein abundances in the YT participants. However, this finding needs to be validated given the limitations listed above. This study also provides unique molecular phenotype data regarding differences in the myofibrillar and sarcoplasmic proteomes between men that differ in age and resistance training experience, and these preliminary data can be used by researchers interested in muscle composition differences between these participants.

## Figures and Tables

**Table 1:** Proteins in the myofibrillar fraction demonstrating a potential long-term training effect.

Protein Symbol	Protein Name	YT Mean ± SD	YU Mean ± SD	OU Mean ± SD
*YT* < *other groups (p* < *0.05)*				
NDUFB8	NADH dehydrogenase 1 beta subcomplex subunit 8	0.5 ± 1.3	4.4 ± 3.9	10.9 ± 7.3
LAMB2	Laminin subunit beta-2	3.7 ± 5.4	12.4 ± 6.2	16.2 ± 12.3
PHB	Prohibitin	6.5 ± 5.6	14.1 ± 5.8	19.6 ± 12.0
CHCHD3	MICOS complex subunit MIC19	13.2 ± 3.6	25.1 ± 12.5	28.6 ± 11.9
DMD	Dystrophin	16.6 ± 6.3	29.2 ± 11.8	50.3 ± 19.8
*YT > other groups (p* < *0.05)*				
MYH16	Putative uncharacterized protein MYH16	487.1 ± 402.4	48.1 ± 117.7	0.0 ± 0.0

Legend: all data are presented as relative expression values (normalized to total spectra).

**Table 2:** Proteins in the sarcoplasmic fraction demonstrating a potential long-term training effect.

Protein Symbol	Protein Name	YT Mean ± SD	YU Mean ± SD	OU Mean ± SD
*YT* < *other groups (p* < *0.05)*				
COQ8A	Atypical kinase COQ8A	5.5 ± 7.1	22.4 ± 16.9	36.6 ± 24.0
*YT > other groups (p* < *0.05)*				
SYPL2	Synaptophysin-like protein 2	79.1 ± 17.5	41.5 ± 26.4	44.1 ± 24.4
PHKG1	Phosphorylase b kinase gamma catalytic chain	22.8 ± 11.0	6.5 ± 8.6	7.4 ± 7.3
HSPA2	Heat shock-related 70 kDa protein	190.4 ± 24.9	145.6 ± 32.7	89.1 ± 100.3
GDI2	Rab GDP dissociation inhibitor beta	61.9 ± 14.4	37.3 ± 18.5	28.0 ± 14.8
ANXA6	Annexin A6	524.8 ± 98.6	409.7 ± 66.7	404.6 ± 68

Legend: all data are presented as relative expression values (normalized to total spectra).

**Table 3:** Proteins in the myofibrillar fraction demonstrating a potential aging effect.

Protein Symbol	Protein Name	YT Mean ± SD	YU Mean ± SD	OU Mean ± SD
*OU* < *other groups (p* < *0.05)*				
ACTA1	Actin, alpha skeletal muscle	5150.3 ± 429.5	5126.8 ± 765.0	4165.7 ± 317.9
ACTC1	Actin, alpha cardiac muscle	4549.8 ± 360.0	3981.9 ± 1404.4	782.3 ± 1452.8
GAPDH	Glyceraldehyde-3-phosphate dehydrogenase	325.9 ± 71.2	318.1 ± 33.0	245.1 ± 41.7
MB	Myoglobin	235.3 ± 96.0	188.3 ± 17.6	136.0 ± 40.1
CSRP3	Cysteine and glycine-rich protein 3	27.7 ± 5.2	29.0 ± 10.0	5.8 ± 9.7
AK1	Adenylate kinase isoenzyme 1	13.9 ± 6.9	13.7 ± 7.7	3.1 ± 4.2
MYBPH	Myosin-binding protein H	34.3 ± 18.2	32.7 ± 27.8	1.3 ± 3.1
*OU > other groups (p* < *0.05)*				
TTN	Titin	3491.7 ± 970.9	3689.1 ± 1566.7	6163.6 ± 611.1
NEB	Nebulin	1087.4 ± 179.1	1185.5 ± 280.2	1572.7 ± 102.0
TNNT1	Troponin T	533.5 ± 81.6	615.4 ± 240.5	958.0 ± 245.9
ATP5PD	ATP synthase subunit d	51.3 ± 19.3	68.7 ± 28.6	115.1 ± 39.6
PLEC	Plectin	43.8 ± 13.0	37.9 ± 19.4	85.9 ± 13.4
HIST3H2BB	Histone H2B type 3-B	19.1 ± 20.0	26.5 ± 18.7	54.2 ± 13.3
DMD	Dystrophin	16.6 ± 6.3	29.2 ± 11.8	50.3 ± 19.8
HIST1H4A	Histone H4	30.5 ± 7.5	33.5 ± 5.0	48.7 ± 12.5
H2AFV	Histone H2A.V	21.3 ± 3.7	24.6 ± 4.2	33.5 ± 4.5
NDUFV2	NADH dehydrogenase flavoprotein 2	17.6 ± 4.5	21.4 ± 4.6	32.4 ± 10.6
DLST	Dihydrolipoyllysine-residue succinyltransferase component of 2-oxoglutarate dehydrogenase complex	7.8 ± 3.8	10.1 ± 2.9	16.8 ± 5.0

Legend: all data are presented as relative expression values (normalized to total spectra).

**Table 4:** Proteins in the sarcoplasmic fraction demonstrating a potential aging effect.

Protein Symbol	Protein Name	YT Mean ± SD	YU Mean ± SD	OU Mean ± SD
*OU* < *other groups (p* < *0.05)*				
KRT9	Keratin, type I cytoskeletal 9	430.4 ± 138.7	392.1 ± 24.9	250.6 ± 61.1
FHL1	Four and a half LIM domains protein 1	322.9 ± 70.9	278.1 ± 32.7	221.9 ± 32.8
KRT2	Keratin, type II cytoskeletal 2	202.9 ± 66.5	185.2 ± 41.3	133.0 ± 31.7
DUSP3	Dual specificity protein phosphatase 3	33.9 ± 11.6	25.3 ± 9.0	8.9 ± 6.9
ANXA2	Annexin A2	37.5 ± 21.7	34.3 ± 16.5	8.2 ± 6.7
GSN	Gelsolin	35.0 ± 20.6	43.7 ± 24.0	7.6 ± 8.6
ACYP2	Acylphosphatase-2	69.1 ± 18.9	43.2 ± 25.7	0.0 ± 0.0
UCHL1	Ubiquitin carboxyl-terminal hydrolase isozyme L1	50.8 ± 29.0	25.7 ± 27.1	0.0 ± 0.0
*OU > other groups (p* < *0.05)*				
ACO2	Aconitate hydratase, mitochondrial	596.0 ± 60.1	601.9 ± 45.4	764.7 ± 141.5
FABP3	Fatty acid-binding protein, heart	474.9 ± 78.3	488.9 ± 84.9	672.2 ± 127.6
MDH1	Malate dehydrogenase, cytoplasmic	386.0 ± 44.5	385.7 ± 61.4	493.6 ± 48.0
HADHA	Trifunctional enzyme subunit alpha	266.0 ± 55.4	287.6 ± 110.4	458.9 ± 141.5
ACADVL	Very long-chain specific acyl-CoA dehydrogenase	214.6 ± 21.5	208.7 ± 60.7	379.2 ± 149.0
HADH	Hydroxyacyl-coenzyme A dehydrogenase	229.1 ± 35.1	228.1 ± 33.9	327.0 ± 69.4
CKB	Creatine kinase B-type	59.8 ± 28.7	69.8 ± 46.2	307.0 ± 157.2
ETFA	Electron transfer flavoprotein subunit alpha	108.5 ± 28.4	120.8 ± 24.2	194.2 ± 61.1
ETFB	Electron transfer flavoprotein subunit beta	74.6 ± 27.7	76.6 ± 28.4	145.9 ± 61.9
ACOT1	Acyl-coenzyme A thioesterase 1	94.9 ± 17.2	92.7 ± 29.2	140.1 ± 31.2
COQ9	Ubiquinone biosynthesis protein COQ9	86.1 ± 16.5	93.3 ± 22.4	127.8 ± 28.1
ES1 homolog	ES1 protein homolog, mitochondrial	55.0 ± 34.0	73.5 ± 31.1	127.3 ± 46.1
HSPA9	Stress-70 protein, mitochondrial	61.3 ± 11.4	62.2 ± 27.1	116.7 ± 47.5
CYCS	Cytochrome c	9.3 ± 10.9	19.1 ± 14.9	59.8 ± 12.7
GPT	Alanine aminotransferase 1	34.7 ± 9.5	30.5 ± 10.3	59.2 ± 14.9
ALDH2	Aldehyde dehydrogenase, mitochondrial	23.5 ± 25.8	21.0 ± 20.6	56.8 ± 24.9
CTSD	Cathepsin D	11.4 ± 8.5	7.6 ± 8.4	53.9 ± 15.4
ALDH5A1	Succinate-semialdehyde dehydrogenase, mitochondrial	24.3 ± 20.6	20.2 ± 15.9	53.3 ± 23.6
AIFM1	Apoptosis-inducing factor 1, mitochondrial	13.2 ± 6.2	8.7 ± 8.9	45.7 ± 26.8
NIPSNAP2	Protein NipSnap homolog 2	15.0 ± 13.6	16.6 ± 16.8	42.8 ± 13.5
FABP4	Fatty acid-binding protein, adipocyte	4.7 ± 5.3	16.0 ± 17.8	35.9 ± 5.9
L2HGDH	L-2-hydroxyglutarate dehydrogenase, mitochondrial	4.8 ± 6.5	6.2 ± 7.2	28.7 ± 22.2
IMPA1	Inositol monophosphatase 1	15.6 ± 8.5	11.3 ± 10.4	26.5 ± 4.3
NDUFB6	NADH dehydrogenase 1 beta subcomplex subunit 6	0.0 ± 0.0	0.0 ± 0.0	25.8 ± 24.4
DCXR	L-xylulose reductase	6.6 ± 7.2	4.7 ± 6.2	24.4 ± 15.3
NDUFB11	NADH dehydrogenase 1 beta subcomplex subunit 11	0.5 ± 1.3	0.0 ± 0.0	22.6 ± 20.5
LRPPRC	Leucine-rich PPR motif-containing protein, mitochondrial	0.0 ± 0.0	0.7 ± 1.8	13.3 ± 12.0
DGLUCY	D-glutamate cyclase, mitochondrial	1.0 ± 1.5	0.0 ± 0.0	12.6 ± 10.3
DCN	Decorin	3.3 ± 5.2	3.9 ± 3.7	11.0 ± 3.4

Legend: all data are presented as relative expression values (normalized to total spectra).

## References

[1] Haun C.T., Vann C.G., Roberts B.M., Vigotsky A.D., Schoenfeld B.J., Roberts M.D. (2019). A Critical Evaluation of the Biological Construct Skeletal Muscle Hypertrophy: Size Matters but So Does the Measurement. Front. Physiol..

[2] Grgic J., Schoenfeld B.J. (2018). Are the Hypertrophic Adaptations to High and Low-Load Resistance Training Muscle Fiber Type Specific?. Front. Physiol..

[3] Evans J.W. (2019). Periodized Resistance Training for Enhancing Skeletal Muscle Hypertrophy and Strength: A Mini-Review. Front. Physiol..

[4] Fry A.C. (2004). The role of resistance exercise intensity on muscle fibre adaptations. Sports Med..

[5] Marzetti E., Calvani R., Tosato M., Cesari M., Di Bari M., Cherubini A., Collamati A., D’Angelo E., Pahor M., Bernabei R. (2017). Sarcopenia: An overview. Aging Clin. Exp. Res..

[6] Trappe S., Gallagher P., Harber M., Carrithers J., Fluckey J., Trappe T. (2003). Single muscle fibre contractile properties in young and old men and women. J. Physiol..

[7] Gelfi C., Vigano A., Ripamonti M., Pontoglio A., Begum S., Pellegrino M.A., Grassi B., Bottinelli R., Wait R., Cerretelli P. (2006). The human muscle proteome in aging. J. Proteome Res..

[8] MacDougall J.D., Sale D.G., Elder G.C., Sutton J.R. (1982). Muscle ultrastructural characteristics of elite powerlifters and bodybuilders. Eur. J. Appl. Physiol. Occup. Physiol..

[9] Toth M.J., Miller M.S., VanBuren P., Bedrin N.G., LeWinter M.M., Ades P.A., Palmer B.M. (2012). Resistance training alters skeletal muscle structure and function in human heart failure: Effects at the tissue, cellular and molecular levels. J. Physiol..

[10] Haun C.T., Vann C.G., Osburn S.C., Mumford P.W., Roberson P.A., Romero M.A., Fox C.D., Johnson C.A., Parry H.A., Kavazis A.N. (2019). Muscle fiber hypertrophy in response to 6 weeks of high-volume resistance training in trained young men is largely attributed to sarcoplasmic hypertrophy. PLoS ONE.

[11] Murgia M., Toniolo L., Nagaraj N., Ciciliot S., Vindigni V., Schiaffino S., Reggiani C., Mann M. (2017). Single Muscle Fiber Proteomics Reveals Fiber-Type-Specific Features of Human Muscle Aging. Cell Rep..

[12] Hody S., Leprince P., Sergeant K., Renaut J., Croisier J.L., Wang F., Rogister B. (2011). Human muscle proteome modifications after acute or repeated eccentric exercises. Med. Sci. Sports Exerc..

[13] Tibana R.A., Franco O.L., Cunha G.V., Sousa N.M.F., Sousa Neto I.V., Carvalho M.M., Almeida J.A., Durigan J.L.Q., Marqueti R.C., Navalta J.W. (2017). The Effects of Resistance Training Volume on Skeletal Muscle Proteome. Int. J. Exerc. Sci..

[14] Esco M.R., Fedewa M.V., Freeborn T.J., Moon J.R., Wingo J.E., Cicone Z., Holmes C.J., Hornikel B., Welborn B. (2019). Agreement between supine and standing bioimpedance spectroscopy devices and dual-energy X-ray absorptiometry for body composition determination. Clin Physiol Funct Imaging.

[15] Haun C.T., Vann C.G., Mobley C.B., Roberson P.A., Osburn S.C., Holmes H.M., Mumford P.M., Romero M.A., Young K.C., Moon J.R. (2018). Effects of Graded Whey Supplementation During Extreme-Volume Resistance Training. Front. Nutr..

[16] Mobley C.B., Haun C.T., Roberson P.A., Mumford P.W., Romero M.A., Kephart W.C., Anderson R.G., Vann C.G., Osburn S.C., Pledge C.D. (2017). Effects of Whey, Soy or Leucine Supplementation with 12 Weeks of Resistance Training on Strength, Body Composition, and Skeletal Muscle and Adipose Tissue Histological Attributes in College-Aged Males. Nutrients.

[17] Roberts M.D., Romero M.A., Mobley C.B., Mumford P.W., Roberson P.A., Haun C.T., Vann C.G., Osburn S.C., Holmes H.H., Greer R.A. (2018). Skeletal muscle mitochondrial volume and myozenin-1 protein differences exist between high versus low anabolic responders to resistance training. PeerJ.

[18] Cohen S., Brault J.J., Gygi S.P., Glass D.J., Valenzuela D.M., Gartner C., Latres E., Goldberg A.L. (2009). During muscle atrophy, thick, but not thin, filament components are degraded by MuRF1-dependent ubiquitylation. J. Cell Biol..

[19] Martin J.S., Kephart W.C., Haun C.T., McCloskey A.E., Shake J.J., Mobley C.B., Goodlett M.D., Kavazis A., Pascoe D.D., Zhang L. (2016). Impact of external pneumatic compression target inflation pressure on transcriptome-wide RNA expression in skeletal muscle. Physiol. Rep..

[20] Hyatt H.W., Toedebusch R.G., Ruegsegger G., Mobley C.B., Fox C.D., McGinnis G.R., Quindry J.C., Booth F.W., Roberts M.D., Kavazis A.N. (2015). Comparative adaptations in oxidative and glycolytic muscle fibers in a low voluntary wheel running rat model performing three levels of physical activity. Physiol. Rep..

[21] Wen Y., Murach K.A., Vechetti I.J., Fry C.S., Vickery C., Peterson C.A., McCarthy J.J., Campbell K.S. (2018). MyoVision: Software for automated high-content analysis of skeletal muscle immunohistochemistry. J. Appl. Physiol. (1985).

[22] Phillips S.M. (2000). Short-term training: When do repeated bouts of resistance exercise become training?. Can. J. Appl. Physiol..

[23] Meijer J.P., Jaspers R.T., Rittweger J., Seynnes O.R., Kamandulis S., Brazaitis M., Skurvydas A., Pisot R., Simunic B., Narici M.V. (2015). Single muscle fibre contractile properties differ between body-builders, power athletes and control subjects. Exp. Physiol..

[24] Bhasin S., Storer T.W., Berman N., Callegari C., Clevenger B., Phillips J., Bunnell T.J., Tricker R., Shirazi A., Casaburi R. (1996). The effects of supraphysiologic doses of testosterone on muscle size and strength in normal men. N. Engl. J. Med..

[25] Tsukamoto S., Shibasaki A., Naka A., Saito H., Iida K. (2018). Lactate Promotes Myoblast Differentiation and Myotube Hypertrophy via a Pathway Involving MyoD In Vitro and Enhances Muscle Regeneration In Vivo. Int. J. Mol. Sci..

[26] Lovering R.M., De Deyne P.G. (2004). Contractile function, sarcolemma integrity, and the loss of dystrophin after skeletal muscle eccentric contraction-induced injury. Am. J. Physiol. Cell Physiol..

[27] Woolstenhulme M.T., Conlee R.K., Drummond M.J., Stites A.W., Parcell A.C. (2006). Temporal response of desmin and dystrophin proteins to progressive resistance exercise in human skeletal muscle. J. Appl. Physiol. (1985).

[28] Tesch P.A., Colliander E.B., Kaiser P. (1986). Muscle metabolism during intense, heavy-resistance exercise. Eur. J. Appl. Physiol. Occup. Physiol..

[29] Costill D.L., Coyle E.F., Fink W.F., Lesmes G.R., Witzmann F.A. (1979). Adaptations in skeletal muscle following strength training. J. Appl. Physiol. Respir. Environ. Exerc. Physiol..

[30] Swaggart K.A., Demonbreun A.R., Vo A.H., Swanson K.E., Kim E.Y., Fahrenbach J.P., Holley-Cuthrell J., Eskin A., Chen Z., Squire K. (2014). Annexin A6 modifies muscular dystrophy by mediating sarcolemmal repair. Proc. Natl. Acad. Sci. USA.

[31] Quattrocelli M., Barefield D.Y., Warner J.L., Vo A.H., Hadhazy M., Earley J.U., Demonbreun A.R., McNally E.M. (2017). Intermittent glucocorticoid steroid dosing enhances muscle repair without eliciting muscle atrophy. J. Clin. Investig..

[32] Gonzalez-Freire M., Semba R.D., Ubaida-Mohien C., Fabbri E., Scalzo P., Hojlund K., Dufresne C., Lyashkov A., Ferrucci L. (2017). The Human Skeletal Muscle Proteome Project: A reappraisal of the current literature. J. Cachexia Sarcopenia Muscle.

[33] Piec I., Listrat A., Alliot J., Chambon C., Taylor R.G., Bechet D. (2005). Differential proteome analysis of aging in rat skeletal muscle. FASEB J..

[34] Kanehisa M., Goto S. (2000). KEGG: Kyoto encyclopedia of genes and genomes. Nucleic Acids Res..

[35] Nagano K. (2015). Alteration of cathepsin-D expression in atrophied muscles and apoptotic myofibers by hindlimb unloading in a low-temperature environment. J. Phys. Ther. Sci..

[36] Lundholm K., Schersten T. (1975). Leucine incorporation into proteins and cathepsin-D activity in human skeletal muscles. The influence of the age of the subject. Exp. Gerontol..

[37] Wiederanders B., Oelke B. (1984). Accumulation of inactive cathepsin D in old rats. Mech. Ageing Dev..

